# RNA-guided gene editing of the murine gammaherpesvirus 68 genome reduces infectious virus production

**DOI:** 10.1371/journal.pone.0252313

**Published:** 2021-06-04

**Authors:** Hui-Chen Chang Foreman, Varvara Kirillov, Gabrielle Paniccia, Demetra Catalano, Trevor Andrunik, Swati Gupta, Laurie T. Krug, Yue Zhang

**Affiliations:** 1 Department of Microbiology and Immunology, Stony Brook University, Stony Brook, New York, United States of America; 2 HIV and AIDS Malignancy Branch, Center for Cancer Research, National Cancer Institute, National Institutes of Health, Bethesda, Maryland, United States of America; Universidade de Lisboa, PORTUGAL

## Abstract

Epstein-Barr virus (EBV) and Kaposi sarcoma herpesvirus (KSHV) are cancer-causing viruses that establish lifelong infections in humans. Gene editing using the Cas9-guideRNA (gRNA) CRISPR system has been applied to decrease the latent load of EBV in human Burkitt lymphoma cells. Validating the efficacy of Cas9-gRNA system in eradicating infection in vivo without off-target effects to the host genome will require animal model systems. To this end, we evaluated a series of gRNAs against individual genes and functional genomic elements of murine gammaherpesvirus 68 (MHV68) that are both conserved with KSHV and important for the establishment of latency or reactivation from latency in the host. gRNA sequences against ORF50, ORF72 and ORF73 led to insertion, deletion and substitution mutations in these target regions of the genome in cell culture. Murine NIH3T3 fibroblast cells that stably express Cas9 and gRNAs to ORF50 were most resistant to replication upon de novo infection. Latent murine A20 B cell lines that stably express Cas9 and gRNAs against MHV68 were reduced in their reactivation by approximately 50%, regardless of the viral gene target. Lastly, co-transfection of HEK293T cells with the vector expressing the Cas9-MHV68 gRNA components along with the viral genome provided a rapid read-out of gene editing and biological impact. Combinatorial, multiplex MHV68 gRNA transfections in HEK293T cells led to near complete ablation of infectious particle production. Our findings indicate that Cas9-gRNA editing of the murine gammaherpesvirus genome has a deleterious impact on productive replication in three independent infection systems.

## Introduction

Herpesviruses are large DNA viruses that cause incurable infections. These viruses typically undergo lytic replication, then establish a lifelong infection predominantly characterized as a quiescent latency program with intermittent reactivation. The two human gammaherpesviruses, Epstein Barr virus (EBV, human gammaherpesvirus 4) and Kaposi sarcoma herpesvirus (KSHV, human gammaherpesvirus 8), are associated with malignant lymphoproliferative diseases and cancers of stromal cells. EBV infects an estimated 90–95% of adults world-wide [1]. The seroprevalence of KSHV ranges from 5% - 90% in adult populations, with highest infection rates in sub-Saharan Africa [2]. People living with HIV and recipients of organ transplants are at highest risk for KSHV and EBV cancers [3].

Novel strategies of intervention are needed to prevent cancer development in people infected with EBV and KSHV. Current therapeutics are limited in efficacy. Nucleoside analogs bind and inhibit the viral DNA polymerase to block DNA replication [4], but do not impact the latency program [5]. Cytotoxic chemotherapies that target proliferating cells are not completely effective in controlling all EBV and KSHV cancers. Immunotherapies that ablate B cells or transfer cytotoxic T cells are costly and experimental. Pomolidomide was recently approved for FDA use against Kaposi sarcoma, but its mechanism of action and utility for other KSHV cancers is unclear [6]. Theoretically, the most effective approach would be the elimination of latent herpesviruses prior to the development of malignancy. For instance, eliminating latent EBV infection in donor bone marrow would reduce the risk of post-transplant lymphoproliferative diseases that arise in ~30% of recipients post-transplant [7].

Murine gammaherpesvirus 68 (MHV68, murid gammaherpesvirus 4) is a natural pathogen of murid rodents with genetic and biologic similarities to the human gammaherpesviruses that is used to study the molecular determinants of pathogenesis in vivo [8, 9]. MHV68 conserves approximately 61 genes among its 80 genes with KSHV [9]. Among the conserved genes that are critical for multiple aspects of virus biology and pathogenesis, MHV68 ORF50 encodes the replication and transcription activator (RTA) that is essential for rhadinovirus replication and reactivation from latency. In the absence of RTA, KSHV is attenuated for early gene expression and viral DNA synthesis, and no infectious particles are produced [10]. A parallel phenotype is observed for MHV68, RTA-null viruses do not grow without complementation by ORF50 expression and are impaired for reactivation from latency [11, 12]. RTA-null viruses are also impaired for latency establishment in the spleens of mice [13, 14].

ORF72 encodes the viral cyclin (v-cyclin), a homolog of mammalian D-type cyclins and is required for viral reactivation from latency in MHV68 and KSHV infections [15]. V-cyclin interacts with several cyclin-dependent kinases (CDKs) resulting in constitutive activation of CDK complexes to enhance proliferation and influence replication in distinct phases of infection [16, 17]. Genetically, the requirement of MHV68 v-cyclin is alleviated in the absence of host CDK inhibitor p18^INK4c^ in vivo, indicating that v-cyclin functions to bypass this CDK inhibitor to promote reactivation from latency [18]. In addition, v-cyclin is an oncogene that promotes KSHV-induced cellular transformation and tumorigenesis by overriding contact inhibition [19]. Expression of MHV68 v-cyclin in early T cells drives lymphoblastic lymphoma [20].

ORF73 encodes the latency-associated nuclear antigen of MHV68 (mLANA) and KSHV (kLANA) that tethers the terminal repeat region of the non-integrated viral genome to the host chromosome and recruits cellular factors to ensure replication and segregation of the replicated viral genomes to daughter cells [5, 9]. In the absence of LANA, KSHV and MHV68 exhibit severe defects in the establishment of latency and dysregulated viral lytic gene expression [21]. The kLANA is functionally interchangeable with mLANA in the context of MHV68 pathogenesis and latency in mice [22]. In addition, mLANA promotes cell survival through inhibition of p53 during lytic viral replication [23]. Consistent with this function of mLANA, kLANA interacts with p53 and the p53-interacting domain is required for establishment of latency and persistent MHV68 infection in mice [24].

The KSHV and MHV68 genomes each harbor two functional lytic origins of replication (oriLyt1 and oriLyt2) [25, 26]. In KSHV, the two oriLyt regions share an almost identical ~1.15 kb sequence and a 600-bp downstream GC-rich repeat sequence. Together, this ~1.7 kb DNA sequence is sufficient to function as a cis signal for replication [26]. Similarly, the oriLyt regions of MHV68 also contain GC-rich repeat sequences [27]. While the two oriLyts are differentially required in several cell lines for lytic replication, both are equally required for efficient latency establishment in mouse spleens following intranasal inoculation of C57BL/6 mice. However, the MHV68 mutant deleted for oriLyt2 was more impaired for ex vivo reactivation from latency [28].

Cas9-gRNA gene-editing technology provides a potentially potent method for disrupting gammaherpesvirus infection and disease. The Cas9-gRNA system consists of a Cas9 endonuclease and a guideRNA (gRNA) which contains a scaffolding region that binds to Cas9 and a 20-nucleotides guide sequence that has homology to a target DNA sequence [29]. The gRNA guides Cas9 to generate a double-strand DNA (dsDNA) break that is typically repaired by non-homologous end joining (NHEJ) that introduces indels into the DNA, potentially knocking out the function of a gene due to a missense or nonsense mutation [30].

Here we examined the impact of this potential therapeutic strategy on MHV68 replication in multiple infection systems. We evaluated the mutational spectra in the targeted loci by a nuclease cleavage assay and deep sequencing. The functional consequence for infectious virus production was determined in fibroblast and epithelial cells permissive for de novo infection and in latent B cells induced to reactivate from latency. These efforts provide a critical step towards the development of a Cas9-gRNA based antiviral therapeutic against gammaherpesvirus infection.

## Materials and methods

### Cells and viruses

NIH3T12 (ATCC CCL-164) and NIH3T3 cells (ATCC CCL-163) were maintained at 37°C in 5% CO_2_ in Dulbecco’s modified Eagle medium (DMEM) supplemented with 8% fetal bovine serum (FBS), 100 U/mL penicillin, 100 mg/mL streptomycin, and 2 μM L-glutamine. X-293T cells (Clontech #632180) and HEK 293T cells were maintained in Dulbecco’s modified Eagle medium (DMEM) supplemented with 10% FBS, 100 U/mL penicillin, 100 mg/mL streptomycin, and 2 μM L-glutamine. A20-HE cells [31] were maintained in RPMI1640 medium containing 10% FBS, 100 U/mL penicillin, 2 μM L-glutamine and 50 μM β-mercaptoethanol. The recombinant MHV68-H2BYFP bacterial artificial chromosome (BAC) was a gift of Dr. Samuel Speck [32].

### Design and synthesis of pLENTICRISPR expressing guideRNAs to MHV68

Three genes and one non-coding element of the MHV68 genome (WUMS strain, NC_001826) were selected as targets for Cas9-gRNA. The 5’ sequences of ORFs 50, 72 and 73 were input into http://crispr.mit.edu [33], and gRNAs 5’ of a PAM site were selected based on a high score, which indicates a low likelihood of off-target binding to the mouse mm9 genome and a 20 bp spacing on opposite strands (Table 1). An approximate 200 bp oligonucleotide geneblock (IDT, San Jose California) was synthesized, which includes the gRNA, a portion of the U6 promoter, and a portion of the gRNA scaffold, flanked by a 5’ NdeI and 3’ EcoRI site. A similar process was used to analyze and clone the non-targeting (NT) gRNAs [34]. None of the NT gRNAs contain PAM sequences. These were digested and ligated into the pLentiCRISPR (Adgene#51760) [35] vector that was digested with EcoRI and NdeI. Clones were identified with PCR screening and verified by sequencing (Stony Brook Genomics Core Facility) and aligned to the *in-silico* design files in Geneious (Version 10.2.4; Biomatters, Auckland New Zealand).

**Table 1:** Guide RNA sequences.

gRNA against MHV68	Sequence of Target Region in MHV68 Genome^a^	Off-Target Score^b^	Construct Name
ORF50 g1	5’ GGATTCCCCTTCAGCCGATAAGG 3’	95	pLentiCRISPR-50g1
ORF50 g2	5’ CAGAAATTCCCTCGTAGTGCAGG 3’	90	pLentiCRISPR-50g2
ORF72 g1	5’ GCACACACAAAACATCCACGTGG 3’	64	pLentiCRISPR-72g1
ORF72 g2	5’ GGATAACAACGTCTTTCCCCTGG 3’	62	pLentiCRISPR-72g2
ORF73 g1	5’ GGCATCCCGGTGGTGGAGGAGGG 3’	71	pLentiCRISPR-73g1
ORF73 g2	5’ AGGTGATGAGGAGTCCAGCCAGG 3’	76	pLentiCRISPR-73g2
OriLyt2 g1	5’ GGGAGCGGGCTGCCCGGCCCGGG 3’	71	pLentiCRISPR-OL2g1
OriLyt2 g2	5’ GCTCCGGGGCCCCGTCCCCCCGG 3’	74	pLentiCRISPR-OL2g2
NT1 ^c^	5’ GCGAGGTATTCGGCTCCGCG 3’	*N/A*	pLentiCRISPR-NT1
NT2 ^c^	5’ GCTTTCACGGAGGTTCGACG 3’	*N/A*	pLentiCRISPR-NT2
NT3 ^c^	5’ GACTCCGGGTACTAAATGTCGT 3’	*N/A*	pLentiCRISPR-NT3
NT4 ^c^	5’ GCTTCTACTCGCAACGTATT 3’	*N/A*	pLentiCRISPR-NT4

^a^ Underlined sequences are PAM sequences.

^b^ Off target scores are the inverse likelihood of off-target binding to the mouse genome.

^c^ Derived from [34].

To produce lentivirus encoding the Cas9-gRNA editing system, X-293T cells at ~80% confluency were transfected with pLentiCRISPR plasmids using the Lenti-X-Packaging Single Shot reagent per manufacturer guidelines (Takara, Mountain View California). After 48 hrs, supernatants were centrifuged at 500 x g for 10 mins and stored at -80°C.

### MHV68 gene editing in cell culture

To generate stable bulk cell lines expressing WT Cas9 and gRNAs, NIH3T3 cells plated at 2x10^5^ cells per well of a 6-well plate one day prior were overlaid with 0.5 mL of the Cas9-gRNA lentivirus stock in 8 μg/mL polybrene. X-293T conditioned media without lentivirus were used as negative controls. The plates were centrifuged at 200 X g for 60 mins at 32°C, then 1.5 mL of 8% FBS-DMEM was added to each well before incubation at 37°C. Puromycin (1.5 μg/mL) was added 24 hrs later, and the media was changed every 3 days for approximately 10 days until all non-transduced cells died and the transduced cells reached confluency and were pooled. Cas9-gRNA expressing NIH3T3 cells (Puro^R^ CRISPR-3T3) were plated at 1X10^5^ cells per well of a 12-well plate and infected the next day with H2B-YFP MHV68 at a low multiplicity of infection (MOI 0.01) in triplicate. Cells were then washed twice with prewarmed PBS before addition of 1 mL of 8% FBS-DMEM media. Cells were collected 96 hpi to analyze viral genomes, infectious particles and to evaluate expression of proteins.

A20-HE cells seeded at 1X10^6^ cells/ml in 12-well plates were nucleofected with 1 μg pLenti-CRISPR plasmids encoding Cas9 and gRNA using methods described before [36]. Cells were selected with 1.0 μg/ml puromycin for approximately four weeks until the resistant Puro^R^ CRISPR-HE cells proliferated at a normal rate. Independent clones were generated with 1–4 clones for each gRNA. Cells seeded at 1X10^5^ cell per well in 12-well plates were either left untreated or treated with TPA at 20 ng/ml for 48 hrs to reactivate latent virus.

HEK293T cells seeded at 1X10^5^ cells per well in 12-well plates one day prior were transfected with 0.25 μg of MHV68-H2BYFP BAC DNA or co-transfected with 0.25 μg MHV68-H2BYFP BAC DNA together with 0.25 μg pLentiCRISPR plasmids using TransIT-LT1 (Mirus) according to the instruction of the manufacturer. Cells were collected 72 hours post transfection to quantitate viral genomes and infectious particles.

### Immunoblot analysis

Infected Puro^R^ CRISPR-3T3 cells as described above were collected in RIPA lysis buffer (150 mM sodium chloride, 1.0% IGEPAL CA-630, 0.5% sodium deoxycholate, 0.1% sodium dodecyl sulfate, 50 mM Tris [pH 8.0]) supplemented with protease inhibitors and PMSF. Protein concentration of the total protein lysate was quantified using Bradford assay (Bio-rad, Hercules, CA) and 40 μg were separated using 4–15% gradient SDS-PAGE and transferred to polyvinylidene fluoride membranes, followed by incubation with antibody to Cas9 (7A9-3A3, Cell Signaling Technology), ORF73 (mLANA) [37], GAPDH (Sigma-Aldrich, St. Louis, MO). Detection was performed with HRP-conjugated anti-mouse or anti-rabbit IgG (Sigma) by enhanced chemiluminescence reagent (ECL, Thermo Scientific). Data was collected with LAS 500 Chemiluminescence Imager (GE Healthcare).

### Quantitation of viral genomes and infectious particles

The copy number of MHV68 normalized to the number of mouse genomes from Puro^R^ CRISPR-HE cells was determined by qPCR. The MHV68 copy numbers in samples were determined using a plasmid standard curve as previously described with primers specific for Orf46 and associated plasmid standard [36]. Similarly, the copy number of mouse genome was determined with primers TertF (5’-GACCTCCTTGTCCTGACCATCTG-3’) and TertR (5’-TCCATTGTGTTCTCTGAGAAGGCA-3), and a plasmid standard containing the amplified product of 101 bp Tert fragment. MHV68 copy number is calculated as the ratio of viral DNA copy number normalized to copies of Tert in the same sample. MHV68 fold increase with TPA is calculated as the fold increase of MHV68 copy number following TPA-treatment over the average of MHV68 copy numbers of the untreated wells of the same clone.

Infectious particles were analyzed by plaque assay. Briefly, NIH3T12 cells were seeded at 100,000 cells per well of a 12-well plate one day prior to infection with a serially diluted, freeze-thaw disrupted cell lysate. Wells were overlaid with 1.5% methylcellulose and incubated at 37°C for 8 days. Cells were washed with PBS, fixed with 100% methanol and stained with 1% crystal violet dye in 20% methanol to visualize plaques in the monolayers and determine PFU/mL.

### Mismatch endonuclease cleavage assay

PCR amplicons of 730–750 bp that encompassed the targeted regions (Table 2) were purified by column filtration, denatured, and self-hybridized prior to digestion with the mismatch endonuclease according to manufacturer instructions (Takara, Mountain View California).

**Table 2:** Amplicon information for endonuclease cleavage assay and NGS.

Amplicon region	Forward (F) and Reverse (R) primer sequences (5’-3’)	Amplicon size (bp)	Expected size of cleavage products (bp)	
			g1	g2
ORF50	F: GCTGGTGAGGCTGGGAAGTTATG	782	~250	~270
	R: GGACAGCCTTGTGGCATTTAATGC		~530	~510
ORF72	F: GTGTGATTAGCACTGGGCGTTTC	652	~240	~260
	R: CACCCCACAACATTCCACCTTC		~410	~390
ORF73	F: GGCAATGGATCAGATGGATTATGCG	747	~270	~260
	R: CCATACTTGAGGGACACCGTACAG		~480	~490

### Next-generation sequencing and analysis of mutations

Genomic DNA isolated from edited or control cells were used as template to amplify a fragment of ~650–780 bp using the primers as listed in Table 2 using Q5 polymerase (NEB). The PCR products were then purified, mixed and diluted to 1 ng/μL for fragmentation and ligation to Index using Nextera XT DNA Library kit (Illumina), then purified with agarose beads. The quantity and fragment range of the samples was verified by Bioanalyzer before 2X75 bp sequencing with NextSeq 500 in the Genomics Core Facility in Stony Brook University.

Data analysis was carried out using Geneious Prime. Sequences were paired and then trimmed using BBDuk at a minimum quality setting of 30 and minimum length of 20 bp. After removal of duplicates, reads were mapped to the reference sequence to identify variations and SNPs. Changes (deletion, insertion or substitution), the number of all the reads encompassing the position of the polymorphism, and the number and frequency of reads of the polymorphism were exported for further analysis in Excel and Prism. The smallest value of each range was depicted for the polymorphisms. Because >90% of reads ranged from 75–76 bp in length, or ~1/10 of the entire amplicon that was randomly fragmented, the co-occurrence of changes in the amplified fragment could not be determined when their distance was larger than 75 bp. Therefore, two different types of calculations were used to assess the frequency of the polymorphism. First, the percent change reflects the frequency of change at the nucleotide(s) position and was calculated by the raw number of reads encoding one specific type of polymorphism and the number of all reads covering the position of this polymorphism. This reflects the frequency of this type of change at a particular position. Second, the percentage of reads containing change provides an estimate of the total frequency of changes within a specific amplicon and was calculated by the number of reads containing any type of change as a percentage of the total reads mapped to this amplicon.

## Results

### Design and construction of lentiviruses expressing guide RNAs targeting at MHV68

**Fig 1:**
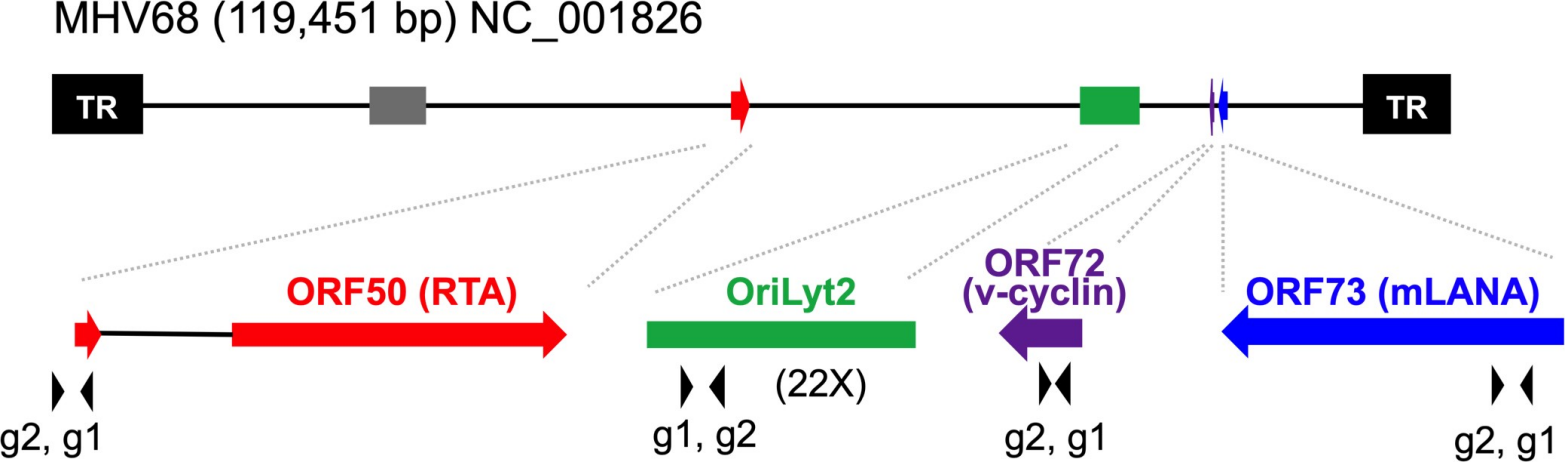
Relative positions and function of viral genes/elements targeted by gRNAs in MHV68 genome. The solid line indicates the whole linear genome of MHV68; the solid black boxes (TR), terminal repeats; gray and green boxes, OriLyt1 and OriLyt2; red arrows, the coding sequences (CDS) of ORF50; purple arrow, ORF72 CDS; blue arrow, ORF73 CDS. The solid black arrowheads under the enlarged ORFs and OriLyt2 indicate the positions of the gRNAs as indicated. Note that ORF50g2 is immediately upstream of the initiation codon of RTA, but within Orf50 Exon 1. The OriLyt2g1 and g2 target the repetitive elements within OriLyt2 that repeat 22 times.

Cas9 generates specific dsDNA breaks 4 nt from the PAM site and through NHEJ repair, an array of substitution, insertion or deletion mutations arise around this position [30]. MHV68 ORFs 50, 72 and 73 are genes conserved with KSHV that have well-characterized functions which promote pathogenesis. The 5’ ends were chosen for gRNA targeting to maximize the potential impact of editing on the protein coding sequence (Fig 1). In addition, a repetitive element within the origin of lytic replication (oriLyt2), was selected as a gRNA target to evaluate the impact of multiple edits in the genome (Fig 1). A pair of gRNA targeting sequences were chosen for each region and they are less than 20 bp apart such that the PAM tri-nucleotides of gRNA targeting sequences were located at the opposite ends away from each other. The original intention was for this pair of gRNA sequences to be used with Cas9_D10A nickase to generate single-stranded nicks for a homology-directed repair method of gene editing. Due to difficulties related to the introduction of a linear oligonucleotide during infection, we instead pursued a NHEJ-directed repair approach using WT Cas9. As controls, non-targeting (NT) sequences were selected from the GECKO CRISPR library [34]. Each gRNA was cloned into the pLentiCRISPR vector to drive concurrent expression of the gRNA with Cas9 and enable puromycin selection [35].

### Stable expression of the CRISPR/Cas9 system in murine NIH3T3 fibroblasts reduces MHV68 production following de novo infection

**Fig 2:**
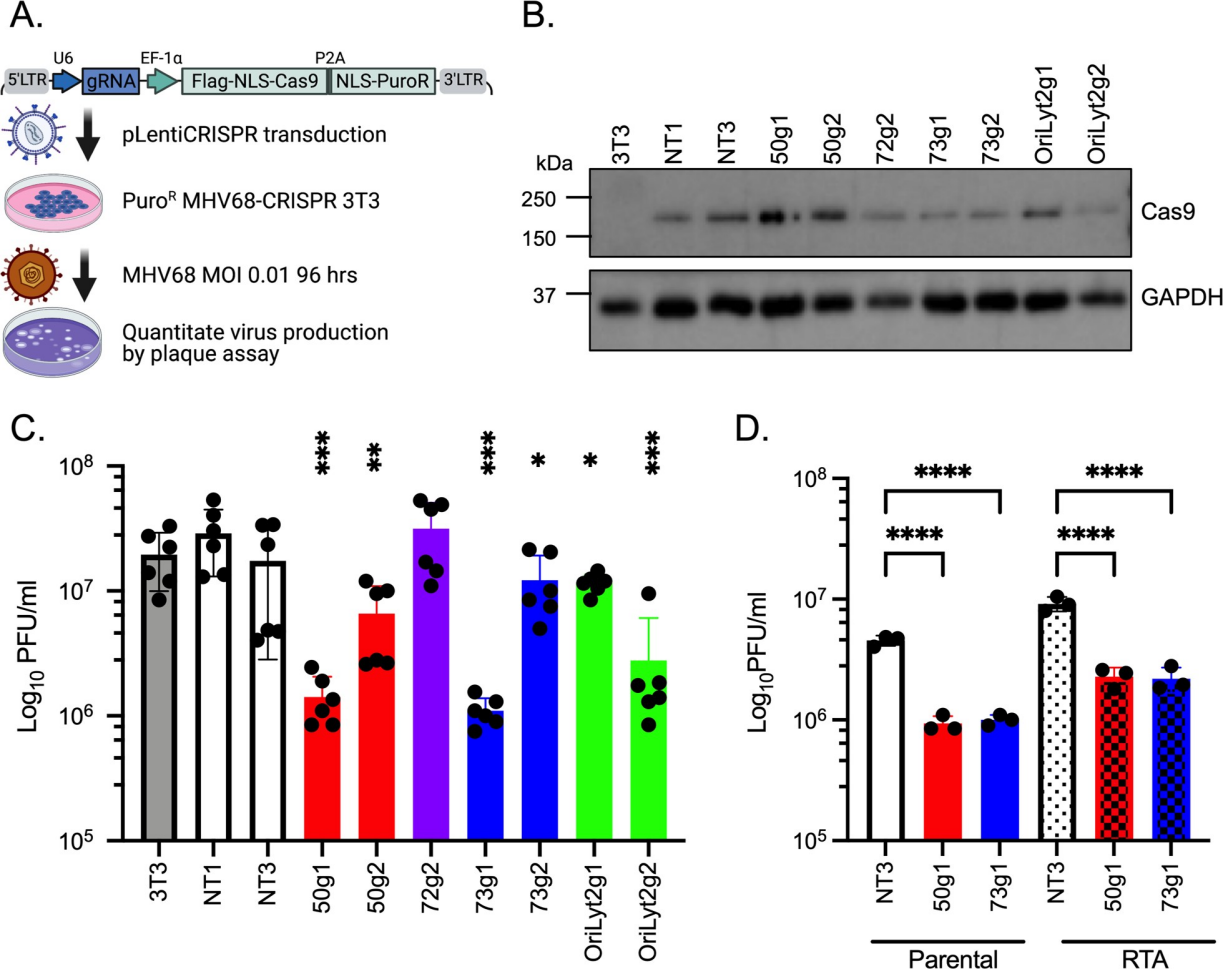
Stable expression of the MHV68 gRNA editing system hinders subsequent viral replication in NIH3T3 cells. **A**. Schematic representation of the approach to evaluate the effect of Cas9 and gRNA-mediated gene editing on lytic MHV68 infection in NIH3T3 cells. Created with BioRender.com. **B.** Expression of Cas9 and cellular housekeeping protein GAPDH in parental fibroblast NIH3T3 cells or the indicated Puro^R^ CRISPR-3T3 cells upon MHV68 infection at MOI of 0.01 for 96 h. **C.** Infectious virus output (PFU/ml) from NIH3T3 or indicated Puro^R^ CRISPR-3T3 cells were determined 96 h after infection with MHV68 at MOI of 0.01. **D.** Infectious virus output from either parental NIH3T12 cells or NIH3T12 cells expressing RTA after infection with viruses prepared from the indicated Puro^R^ CRISPR-3T3 cell lines. Ordinary one-way ANOVA followed by Dunnett’s multiple comparison test with NT1 was used to determine significance as indicated: *, p<0.05; **, p<0.01; ***, p<0.001; ****, p<0.0001. Results shown are combined from two independent experiments.

Loss of ORF50 and ORF73 function upon mutation in MHV68 is expected to impact virus replication. To test whether the Cas9-gRNA system interferes with de novo virus infection, NIH3T3 cells stably transduced with lentiviruses to express Cas9 and individual gRNAs (Puro^R^ CRISPR-3T3) were infected with wild type MHV68 at a low multiplicity of infection (MOI) of 0.01 plaque forming units (PFU) per ml (Fig 2A). Cas9 was detected in the stable transductants (Fig 2B). As expected, NIH3T3 cells transduced with non-targeting gRNAs (NT1 or NT3) did not interfere with virus replication at 96 hpi (Fig 2C). Consistent with the observation that v-cyclin is not required for MHV68 replication in fibroblasts [15], viral titers from 72g2 Puro^R^ CRISPR-3T3 cells were similar to that from NT1 Puro^R^ CRISPR-3T3 cells (Fig 2C). However, lower levels of infectious virus were observed in all other Puro^R^ CRISPR-3T3 cells. 50g2, 73g2 and OriLytg1 cells had a moderate 0.4 to 0.7 log decrease in infectious virus production (Fig 2C). A drop in virus production by more than one log was observed for 50g1, 73g1 and OriLytg2 Puro^R^ CRISPR-3T3 cells (Fig 2C). Expression of Cas9 and gRNAs targeting three independent regions of the MHV68 genome at the onset of de novo infection reduce virus output in fibroblast cells.

Since 50g1 targets the first exon of ORF50 CDS [38, 39], gene editing in this region was expected to result in loss of RTA function, which is required for lytic replication. Thus, one possibility for the reduction of infectious particles as enumerated by plaque assay is that ORF50-edited viruses were produced yet could not lead to plaques due to loss of RTA. To address this, NIH3T12 cells expressing the wild type RTA protein that fully restore the replication of MHV68 mutants lacking ORF50 [12] were used to measure infectious virus produced from the 50g1 Puro^R^ CRISPR-3T3 cells. Virus produced from NT3 and 73g1 Puro^R^ CRISPR-3T3 cells were used as controls. As observed in Fig 2C, a significant reduction in infectious virus was produced from either 50g1 or 73g1 Puro^R^ CRISPR-3T3 cells, compared to that from NT3 cells when titered on parental 3t12 cells (Fig 2D, first three lanes). In RTA-expressing 3T12 cells, slightly higher viral titers were generated consistent with heightened RTA expression and immediate early lytic gene transactivation functions (Fig 2D, last three lanes). However, RTA-expression did not fully restore infectious titers for the inoculum produced from the 50g1 Puro^R^ CRISPR-3T3 cells (Fig 2D). These results indicate that the gRNA targeting 50g1 effectively impairs virus production altogether, the reduction was not due to mutations in the lytic transactivator gene that would preclude enumeration by plaque assay.

### Gene editing in the context of lytic replication

**Fig 3:**
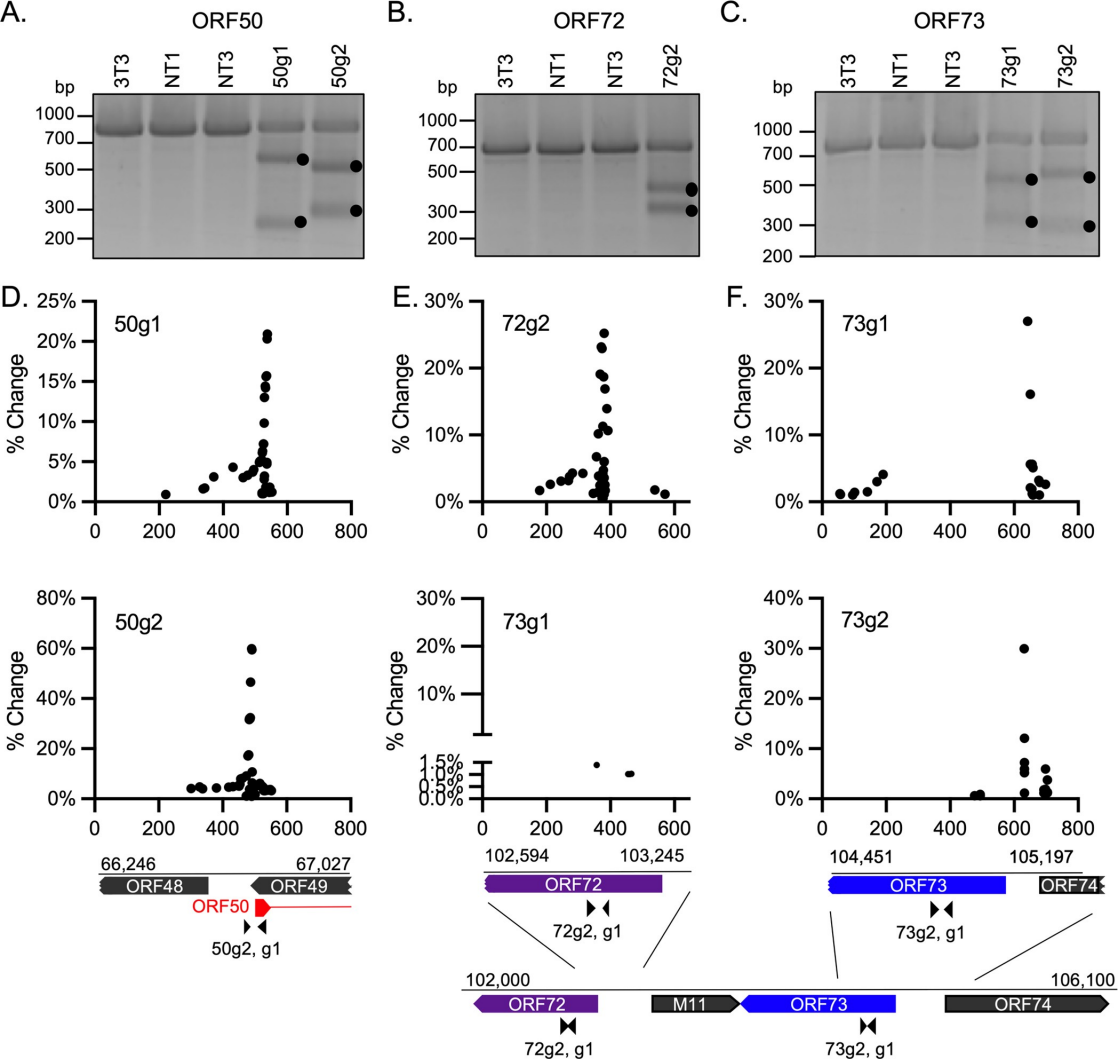
Evaluation of gene editing by mismatch endonuclease cleavage and targeted next generation sequencing. **(A-C)** Amplicons from the indicated genomic regions were generated from parental NIH3T3 (3T3) or the indicated Puro^R^ CRISPR-3T3 cells 96 hpi (MOI of 0.01) and subjected to mismatch endonuclease cleavage. The black dots indicate the cleaved DNA fragments. **(D-F)** Amplicons from the indicated genomic regions were generated from the indicated Puro^R^ CRISPR-3T3 cells 96 hpi (MOI of 0.01) and analyzed for mutations by next-generation sequencing (NGS). Reads obtained from NGS were mapped to the respective amplicon sequence according to MHV68 reference sequence (NC_001826) and the percentage of reads containing each specific change was determined. Each dot represents one specific change observed at the indicated position and the percent change (% change) indicates the percentage of reads containing a unique change among all reads encompassing the position of the change. The gene structure and genomic coordinates of the amplicon is depicted below the graphs. Small triangles indicate gRNA positions. Arrows indicate direction of ORFs and broken ends depict a portion of the incomplete CDS.

To assess gene editing, DNA fragments encompassing the gRNA targeting sequences were PCR amplified using high fidelity polymerases and subject to mismatch endonuclease cleavage analysis. In this assay, amplicons are denatured and rapidly rehybridized such that DNA hybrids with unpaired bases due to indels are substrates for endonuclease cleavage. Though the Cas9-mediated dsDNA break is at -4 position from the PAM site, NHEJ repair results in random incorporation of indels around the break point [30]. As expected, amplicons prepared from infected parental NIH3T3 cells or NT1, NT3 Puro^R^ CRISPR-3T3 cells were not cleaved (Fig 3A–3C). However, amplicons prepared from infected 50g1 and g2 (Fig 3A), 72g2 (Fig 3B), and 73g1 and g2 (Fig 3C) exhibited DNA-fragmentation patterns consistent with the prediction according to the relative gRNA position (Table 2). For 72g2 Puro^R^ CRISPR-3T3 cells, virus replication was not impacted (Fig 2C), but mismatch endonuclease cleavage analysis indicated the presence of gene editing (Fig 3B). The repetitive nature and the high-GC content OriLyt2g1 and g2 genomic regions precluded this analysis. Taken together, gRNAs directed to ORFs 50, 72, and 73 led to gene editing at the expected sites in the MHV68 genome with variable impacts on infectious virus production.

We next analyzed the spectra of mutations in the genomic regions of MHV68 after exposure to the Puro^R^ CRISPR-3T3 cells. A change is defined as any unique difference in comparison to MHV68 reference sequence; this includes a deletion, substitution or insertion of one or more nucleotides. No changes were identified in amplicons prepared from infected parental 3T3 cells or control Puro^R^ CRISPR-3T3 cells expressing NT1 or NT3 (Table 3). Of the amplicons prepared from cells expressing MHV68 gRNAs, variable numbers of changes were identified. As detailed in Material and Methods, due to the small stretch of reads obtained from NGS, two methods were used to quantify the changes identified. First, as shown in Fig 3D and 3E, the location and the frequency of specific changes were depicted. Secondly, changes were also summarized in Table 3 according to the type of change, either insertion, deletion or substitution. In amplicon ORF50 from infected 50g1 Puro^R^ CRISPR-3T3 cells, >50% of the reads were annotated as containing at least one type of change with the majority of the changes noted as deletions (Table 3). A striking 100% of the reads of ORF50 amplicons generated from infected 50g2 Puro^R^ CRISPR-3T3 cells contained changes with ~95% of the changes defined as deletions (Table 3). These changes clustered around the expected dsDNA break points (Fig 3D). The number of changes gradually diminished to the left of this center but stopped abruptly to the right. Most changes to the left side were located between the coding regions of ORF48 and ORF49 (Fig 3D). In amplicon ORF72 obtained from infected 72g2 Puro^R^ CRISPR-3T3 cells, ~53% of reads contained changes, the majority were deletions (Table 3). Again, the changes clustered at the expected break point, and distributed more on the left side (Fig 3E, top panel). Changes were not observed in a short stretch of DNA at the beginning the ORF72 CDS. In contrast, only 3.89% and 1.8%, respectively, of the total reads of ORF73 amplicons obtained from 73g1 or 73g2 Puro^R^ CRISPR-3T3 cells post infection contained changes (Table 3). The majority of the changes observed were deletions, followed by nucleotide substitutions and insertions. Most of these changes were located between the ORF73 and ORF74 CDS (Fig 2F). The rarity and the location of the edits observed in the infected 73g1 or g2 Puro^R^ CRISPR-3T3 cells could be attributed to low editing efficiency or the elimination of viruses following edition in the region.

**Table 3:** NGS results for amplicons prepared from infected Puro^R^ CRISPR-3T3 cells.

Amplicon region	gRNA targeting region:	# of Total reads^a^ in corresponding amplicon	% of Reads containing change	Type of change		
				Deletion	Insertion	Substitution
ORF50	ORF50g1	362,235	51.23%	47.97%	0.19%	3.17%
ORF50	ORF50g2	360,475	100% ^b^	94.98%	2.63%	4.86%
ORF50	NT1	132,098	ND ^c^			
ORF50	NT3	116,740	ND			
ORF72	ORF72g2	343,432	52.83%	49.59%	0.56%	2.36%
ORF72	NT1	94,885	ND			
ORF72	NT3	164,758	ND			
ORF73	ORF73g1	409,964	3.89%	3.75%	undetected	0.14%
ORF73	ORF73g2	130,093	1.80%	1.62%	0.03%	0.15%
ORF73	NT1	100,129	ND			
ORF73	NT3	88,280	ND			
Evaluation of off-target effect:						
ORF**72**	ORF**73**g1	327,707	0.49%	0.33%	undetected	0.17%

The number of reads that mapped to the reference sequence.

^b^More reads of Amplicon-ORF50 prepared from ORF50g2 Puro^R^ CRISPR-3T3 cells contained changes than the total number of reads mapped to the region. Some reads may contain both deletions and substitutions, but the program does not distinguish reads containing two (or more) changes from those containing only one type of change.

^c^ND: none detected.

NGS also provided an opportunity to evaluate the presence of rare off-target editing events or changes presented further away from the expected breaking points. Of all the possible amplicons prepared from samples that expressed irrelevant targeting gRNA (e.g., amplicon ORF50 from 73g1 Puro^R^ CRISPR-3T3 cells), only amplicon ORF72 obtained from 73g1 Puro^R^ CRISPR-3T3 cells contained edits (Fig 3E, bottom panel). As expected, the events were rare such that only 0.49% of the reads carried changes (Table 3). Of note, 72g2 and 73g1 are less than 1800 bp away. Overall, the NGS results confirm that our Cas9-gRNAs system led to specific editing of viral gene targets during an active viral infection of NIH3T3 fibroblast cells.

### Stable expression of the CRISPR/Cas9 system reduces MHV68 reactivation

**Fig 4:**
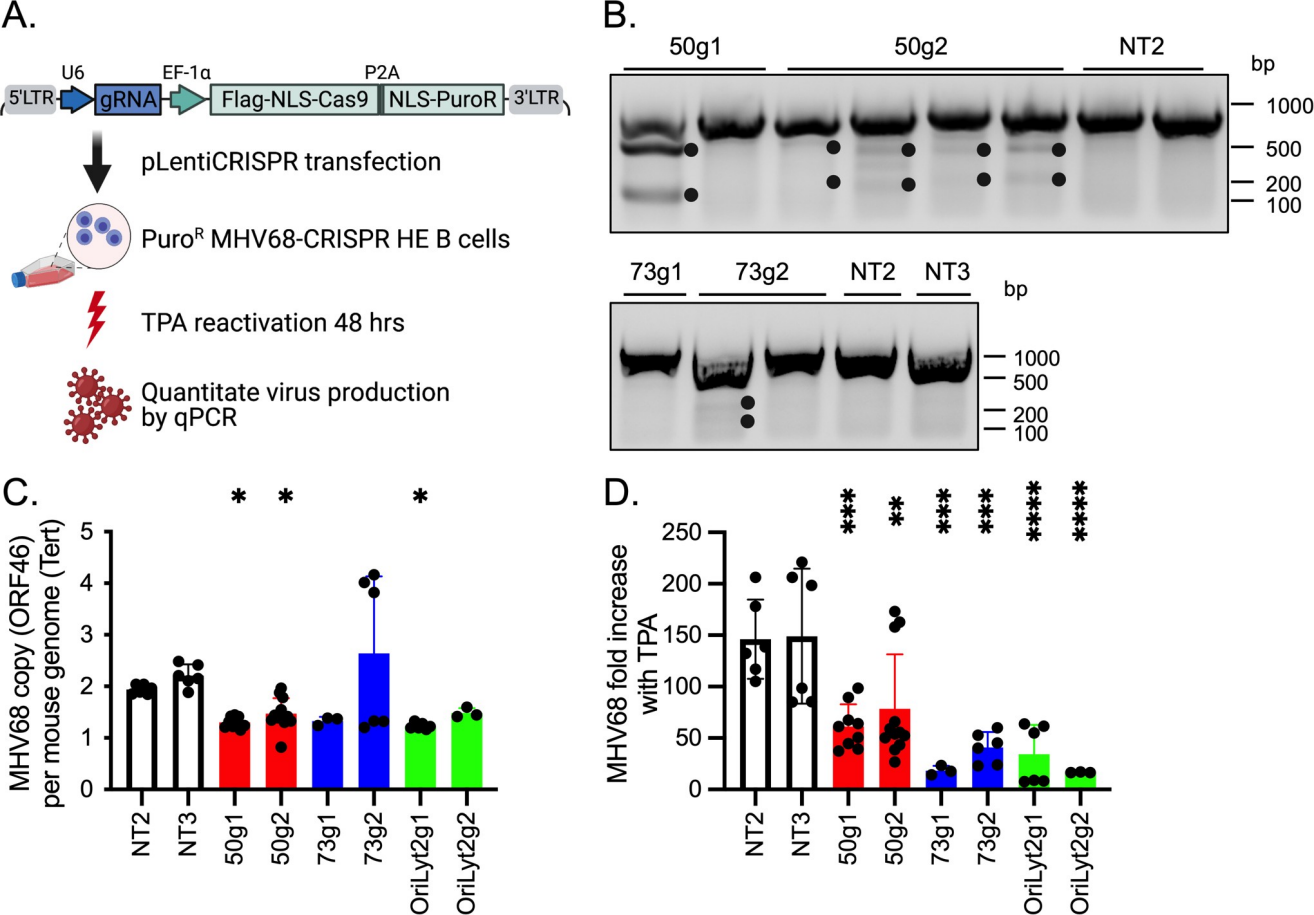
Stable expression of the MHV68-specific Cas9-gRNA system reduces latent MHV68 copy number per cell and hinders subsequent viral reactivation in A20-HE cells. **A.** Schematic representation of the approach to evaluate the effects of Cas9-gRNA system mediated gene editing on latent MHV68. Created with BioRender.com. **B.** Mismatch endonuclease cleavage of amplicons ORF50 (top) and ORF73 (bottom) prepared from the indicated Puro^R^ CRISPR-HE clones. With the same gRNA, each lane represents an independent clone. **C**. MHV68 viral load determined by qPCR with MHV68 ORF46 and normalized to mouse Tert. **D.** Reactivation of latent MHV68 by TPA treatment. Viral copies were determined by qPCR and normalized to mouse Tert. The fold change in viral copy number after TPA treatment relative to the average viral copy number in basal, uninduced conditions of the same clone are plotted. For C and D, results were compiled from four experiments containing independent clones of Puro^R^ CRISPR-HE cells described in (B), with six repeats for 50g1 clone 1, NT2 and NT3, and three repeats for all other clones. Ordinary one-way ANOVA followed by Dunnett’s multiple comparisons with NT3 (C) and NT2 or NT3 (D) were used to determine statistical significance as indicated. *: p<0.05; **: p<0.01; ***: p<0.001 and ****: p<0.0001.

Next, we evaluated the capacity of this CRISPR-Cas9 system to edit latent viral genomes. The A20-HE B-cell line is latently infected with reactivation-competent MHV68 [31]. A20-HE cells were nucleofected with pLentiCRISPR plasmids encoding gRNA targeting MHV68 or NT controls and 1–4 clones of Puro^R^ CRISPR-HE cells expressing each gRNA were selected (Fig 4A). The mismatch endonuclease cleavage analysis of amplicons prepared from individual Puro^R^ CRISPR-HE clones verified gene editing in the expected genomic region, albeit at variable levels between clones (Fig 4B). Next, the copy number of viral genomes was determined using primers specific to MHV68 ORF46, a region not related to the gRNA targets [36]. The copy number of mouse genome was determined using primers for the host Tert sequence. Using DNA isolated from NT2 or NT3 Puro^R^ CRISPR-HE clones, the mean ratio of the copy number of ORF46 to Tert was close to 2. Considering that each normal cell contains two copies of Tert, this result indicates that in these cells, an average cell contains about 4 copies of the virus (Fig 4C). In multiple clones of 50g1, 50g2 and O2g1 Puro^R^ CRISPR-HE cells, the ORF46/Tert ratio was reduced down to an average of 1.2 to 1.5 (Fig 4C). These results indicate that the MHV68-targeting Cas9-gRNA system is also suitable to edit the latent viral genomes of B cells. To determine the impact of such editing on viral reactivation from latency, the fold increase of viral genomes was examined after stimulating the cells to reactivate with TPA. Reactivation varied between clones and experiments, but a clear trend of lower reactivation was observed in clones expressing MHV68 targeting gRNAs compared to control NT-gRNAs (Fig 4D). In NT2 and NT3 Puro^R^ CRISPR-HE cells, viral copies increased 146- and 149-fold following TPA stimulation, respectively (Fig 4D). In contrast, only a mean 61- and 78-fold increase was observed in respective 50g1 and 50g2 Puro^R^ CRISPR-HE cells, indicating a 50% reduction in viral reactivation (Fig 4D). A more dramatic decrease in reactivation was observed in 73g1 and g2 Puro^R^ CRISPR-HE cells such that the viral genome copy number only increased by a respective average of 18- and 41-fold (Fig 4D). A significant decrease in viral reactivation was also evident in OriLyt2g1 and g2 Puro^R^ CRISPR-HE cells where an average of 34- and 17-fold induction in viral copy number occurred (Fig 4D). In sum, Cas9-gRNA directed gene editing decreases latent viral load and reduces the efficiency of MHV68 reactivation in latent B cells.

### Near complete blockage of MHV68 multiplication in 293T cells following transfection of multiple pLentiCRISPR-MHV68gRNA plasmids

**Fig 5:**
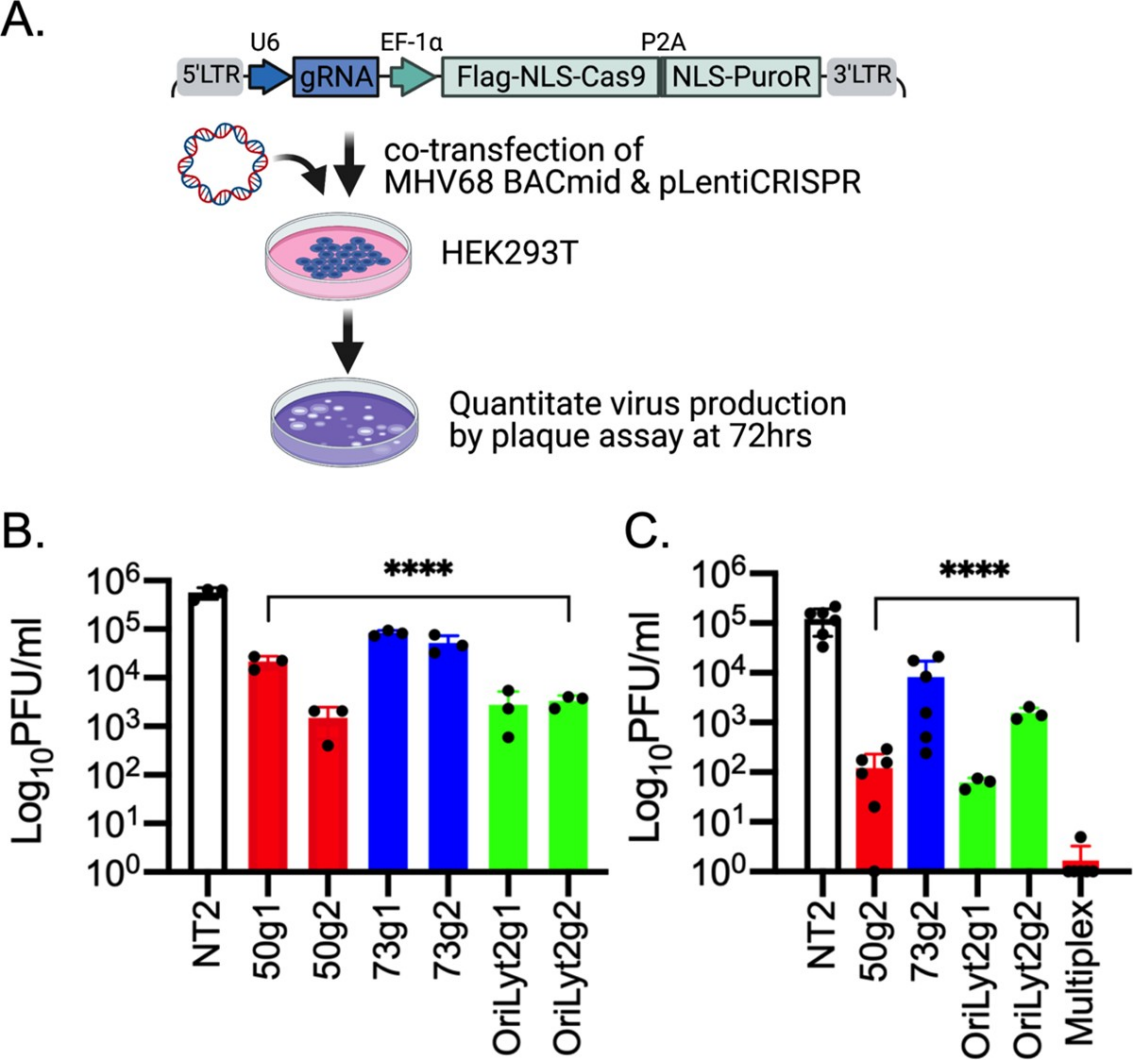
Multiplex gRNAs knock-down of MHV68 virus production. **A.** Schematic representation of the approach to evaluate the effects of Cas9-gRNA-mediated gene editing in 293T cells upon co-transfection of MHV68 BACmid DNA with pLentiCRISPR plasmid(s). Created with BioRender.com. **B.** Infectious virus output analyzed 72 hrs post-transfection with the indicated gRNAs in single. Data is representative of two independent experiments performed in biological triplicate. **C.** Infectious virus output analyzed 72 hrs post-transfection with single or multiple gRNAs. Multiplex samples delivered 50g2, 73g2, OriLyt2g1 and g2. Ordinary one-way ANOVA followed by Dunnett’s multiple comparisons to NT2 was used to determine significance as indicated: ****, p<0.0001. Results are the compilation of two independent experiments, each with three biological replicates.

Our results indicated that the expression of Cas9 and MHV68-specific gRNAs led to editing of the viral genome during lytic infection and latency that impacted virus production. However, the efficiency of edits and reduction in virus output was variable. We postulated that gene editing and double strand breaks at multiple locations on each viral genome would have a greater impact. To evaluate the impact of a multiplex approach, the effect of the gRNAs was tested in singlet and then in combination upon transfection with the infectious MHV68 bacmid genome in HEK293T cells (Fig 5A). Co-transfection of the MHV68 bacmid together with plasmid encoding control gRNAs NT2 resulting in an average viral titer of 5.6 X10^5^ PFU/ml (Fig 5B). In comparison, co-transfection with plasmids encoding MHV68 targeting gRNA resulted in a 1–3 log decrease in infectious virus production (Fig 5B). 50g2 tended to be more effective in reducing virus multiplication; an average of only 1.5X10^3^ PFU/ml were produced in HEK293T cells co-transfected with plasmid encoding 50g2 and the MHV68 bacmid, while the average viral titer was 2.1X10^4^ PFU/ml for 50g1 (Fig 5B). In comparison, the 73g1 and g2 gRNAs led to a moderate decrease to 8.4 X10^4^ and 5.2 X10^4^ PFU/ml, respectively (Fig 5B). OriLyt2g1 and g2 had similar impacts on infectious virus production, reducing yield to 2.8 X10^3^ and 3.4 X10^3^ PFU/ml, respectively. Next, 50g2, 73g2, OriLyt2g1 and g2 were chosen to evaluate the impact of combinatorial gRNA targeting on virus production. MHV68 bacmid co-transfection with plasmid encoding control NT2 yielded 1.2 X10^5^ PFU/ml. A 2–3 log decrease was observed upon single administration of 50g2, 73g2, OriLyt2g1 or g2 (Fig 5C). However, multiplex administration of these MHV68-targeted gRNAs led to a striking reduction to 1–2 PFU/ml of infectious particles, at the cusp of the limit of detection. This striking ~5 log decrease in virus production suggests that simultaneous editing of different regions of the viral DNA leads to additive repression of virus replication.

## Discussion

We report that Cas9-gRNA gene editing technology reduced MHV68 replication in three distinct cell culture systems that represent both lytic and latent models of infection. Using a mismatch endonuclease assay followed by NGS, gene editing was confirmed at the predicted locations in the viral genome. Gene editing did not entirely eliminate virus replication, but virus replication was significantly impaired as evidenced by a decrease in viral DNA load or reduction in infectious particle production across cell systems. Significantly, multiplex gRNA delivery resulted in the largest reduction in virus production by as much as ~5 logs per ml of culture.

The NGS and mismatch endonuclease cleavage analyses demonstrated gene editing by the MHV68-targetted gRNAs. In the Puro^R^ CRISPR-3T3 and the Puro^R^ CRISPR-HE cells, the mismatch endonuclease cleavage analysis indicated that the coding regions of the viral genes were specifically targeted by their respective gRNAs (Figs 3A–3C and 4B). NGS analysis of the infected CRISPR-3T3 cells indicated that 50g2 led to the highest frequency of edits, followed by 50g1 and 72g2; while editing directed by 73g1 and 73g2 was less effective (Table 3). Even so, both 50g1 and 73g1 led to similar reductions in virus output in NIH3T3 cells that exceeded the reduction by 50g2 and 73g2 (Fig 2C). This apparent lack of correlation between the efficiency of editing and the impact on lytic replication of particular gRNAs might involve PCR bias inherent in the initial amplification and NGS library construction and sequencing. Another consideration is that the mismatch endonuclease cleavage and NGS analyses only assess the viral genomes that remain at the time of sampling. In other words, if the Cas9-gRNA gene editing leads to decreased viral genome replication via loss of lytic gene function, a cis replication element or direct damage to template genomes, the edited genomes will be underrepresented in the DNA from the infected cell culture.

The apparent escape from editing might also reveal novel biology. ORF50g2 was designed to knock-out exon 1 but curiously did not dramatically reduce lytic replication in spite of a high efficiency of editing. Splicing in the ORF50 locus is complex, characterized by alternate transcript isoforms that originate from multiple upstream promoters [38, 39]. Characterization of 50g2-edited mutants might reveal novel ORF50 transcripts.

Although a reduction in the average number of latent viral genomes per cell was observed in the MHV68 gRNA Puro^R^ CRISPR-HE cells in comparison to those NT ones (Fig 4C), lytic replication in cell culture system was the primary readout in our studies. These systems may not be sufficient to analyze the full biological impact of edits in ORF72 (v-cyclin) or ORF73 (mLANA). While v-cyclin has been shown previously to have some role in acute virus replication, the major phenotype of v-cyclin mutants is a defect in reactivation from latency in splenocytes of infected mice [15]. The mLANA knockout virus exhibits altered gene expression during lytic replication [40], but mLANA mutants are most severely compromised in the establishment of latency [41]. A recent study evaluating the effects of Cas9-gRNA in cells carrying latent KSHV also chose LANA as a target and observed a partial decrease in viral genome copies [42]. They reasoned that inefficient delivery of Cas9-gRNA system to B cells was a major hurdle [42]. Future studies of latency in the B cell reservoir in mice will require novel strategies and/or transgenic mice to deliver the gRNA and Cas9 components.

An additional consideration for herpesvirus editing is the higher order chromatinized structure of the viral genome that may preclude gRNA recognition and accessibility for Cas9 cleavage. ORF50 encodes the immediate early RTA protein and its transcription in the early stages of de novo infection or reactivation would likely provide an open chromatin structure that is more sensitive to gene editing. On the other hand, since mLANA expression is required to maintain the viral genome during latency when B cells proliferate, the ORF73 region would be an ideal locus to target during latency. Interestingly, we observed a more severe reduction in viral load in the context of reactivation than during long-term steady state latency for many gRNAs. Cas9-gRNA editing of the EBV genome and viral clearance has been demonstrated for latently infected tumor cells. Indeed, Wang and Quake observed complete elimination of the EBV from latent Raji cells upon CRISPR-gene editing [43], but Cas9-gRNA appeared inefficient at targeting quiescent HSV-1 genomes [44]. Aubert et al. recently reported that AAV-delivered meganucleases were superior to CRISPR in mediating efficient gene editing and elimination of latent HSV in vivo [45]. Furthermore, HSV gene edits driven by meganucleases were increased in the context of a histone deacetylase inhibitor [46]. While meganucleases might also be considered for elimination of latent gammaherpesvirus, histone deacetylase inhibitors might improve the efficacy of editing latent genomes by CRISPR.

The impact of gene editing on MHV68 was variable within and across cell culture systems. This may relate to Cas9 expression levels that vary in copy number or with the site of lentiviral integration. Genetic drift occurs in cell culture and may be exacerbated when drug-selection is applied. Due to the high GC-content and repetitive nature of the oriLyt2 sequence, we failed to consistently amplify this genomic region, but a reduction of viral titers was observed in all three cell systems with gRNA targeting to this region. It remains to be determined if virus reduction was due to the loss of a cis-element that contributes to lytic DNA replication or if multiple edits in close proximity triggered a DNA damage response that results in death of both the cell and the virus [46, 47].

HEK293T cells provided the most robust readout for changes in virus output and enabled combinatorial gRNA analysis. We delivered infectious viral genomes along with Cas9-gRNA constructs to target single or multiple viral targets. In addition to the nearly 100% transfection efficiency that is achieved in HEK293T cells, cytoplasmic delivery of the transfected material may provide gRNAs unhindered access to naked genomes. This contrasts with the higher order chromatinized genomes in latent B cells and the encapsidated genomes upon de novo infection of fibroblast cells. Multiplex gRNA exposure to the MHV68 genome led a potent additive amelioration of virus production in HEK293T cells. A multiplex targeting strategy was found to be more effective than single gRNA target in decreasing EBV genome copy numbers in Raji lymphoma cells [43]. A mixture of gRNAs targeting several viral genes was shown to be more effective in decreasing viral replication in other herpesvirus models [48]. Taken together, once individual gRNAs are validated, a multiplex targeting strategy should be considered to physically broaden the impact of dsDNA breaks and the editing of viral genes and genomic regions that drive pathogenesis.

Cas9-gRNA have been applied to target latent viral infections including herpes simplex virus type 1, human cytomegalovirus, EBV, KSHV and HIV in cell culture [42–44, 49]. These reports showcased the potential therapeutic value of targeted gene editing in treating lifelong viral infections. A number of critical barriers to the efficient implementation of antiviral gene editing technology remain. First, an efficient method for *in vivo* delivery of nucleases like Cas9 to target reservoirs of latency is required [50]. Second, the efficiency of gene editing is constrained by the chromatinization of the targeted region [51]. Third, off-target effects must be minimized.

Here we report the first application of a Cas9-gRNA gene editing system to directly target and edit MHV68 in the context of virus elimination for a therapeutic purpose. The genes and functional OriLyt elements in MHV68 have direct homologs with analogous functions in KSHV infection. The results presented here provide a foundation for future evaluation in a small animal pathogenesis model. This will enable pre-clinical evaluation of the feasibility of delivery to latent B cell reservoirs, the efficacy of reducing viral load, and safety of a gene editing system towards the eradication of gammaherpesviruses in vivo.

## Supporting information

S1 Raw images The original blots of Fig 2B and original gels of Figs 3A–3C and 4B as indicated. Samples and size markers of the original blots or gels were labeled according to the descriptions of the final figures shown in the main text and unused lanes were indicated with “X”.
(PDF)
