# Supplementary material for: RNA-guided gene editing of the murine gammaherpesvirus 68 genome reduces infectious virus production
Source: PLoS One. 2021 Jun 4;16(6):e0252313. doi: 10.1371/journal.pone.0252313 (PMC8177658; doi:10.1371/journal.pone.0252313)

Final Figure 2B

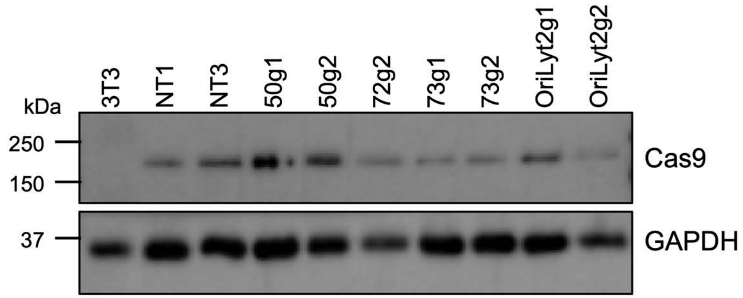

Original blots

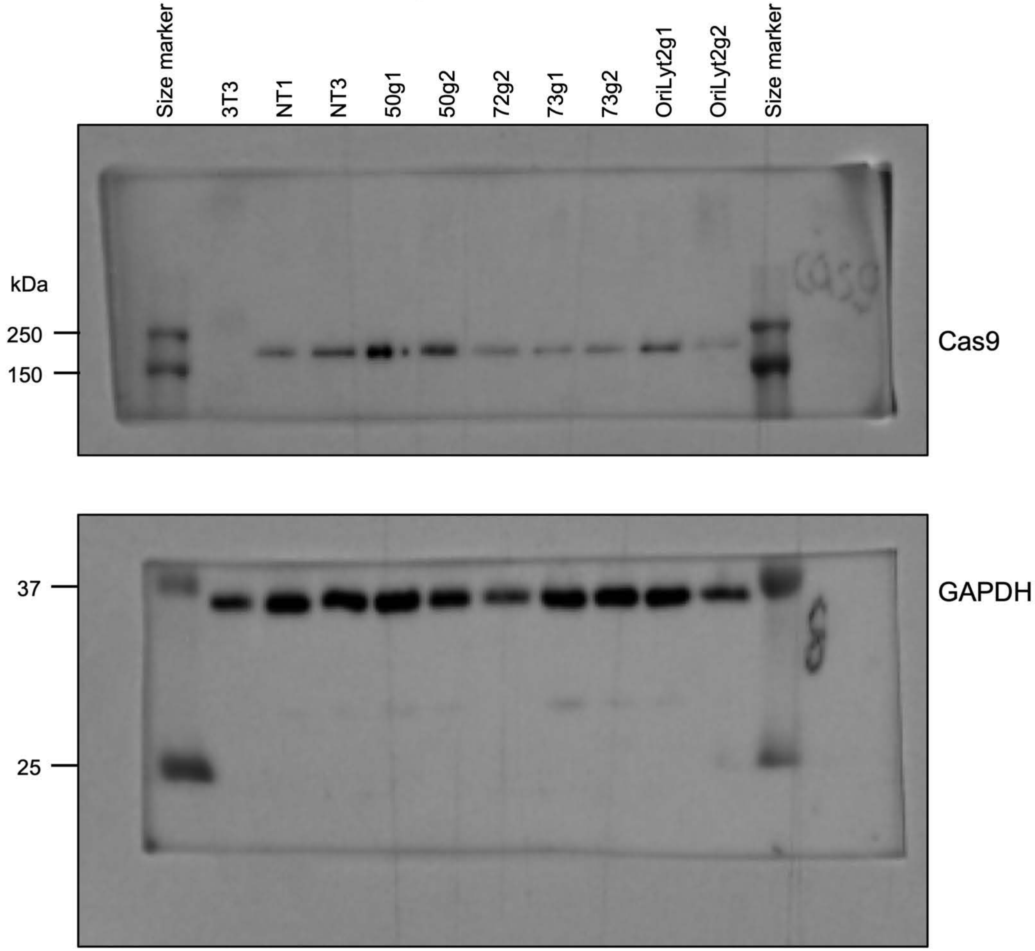

Final Figure 3A-C

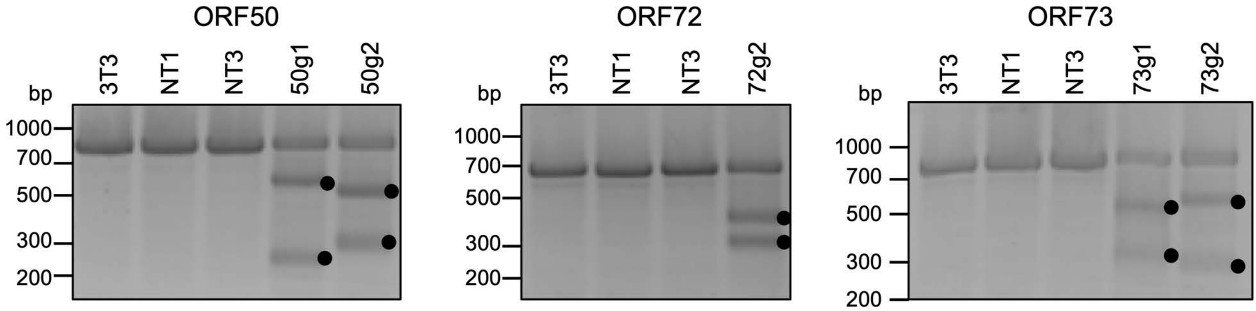

Original gels

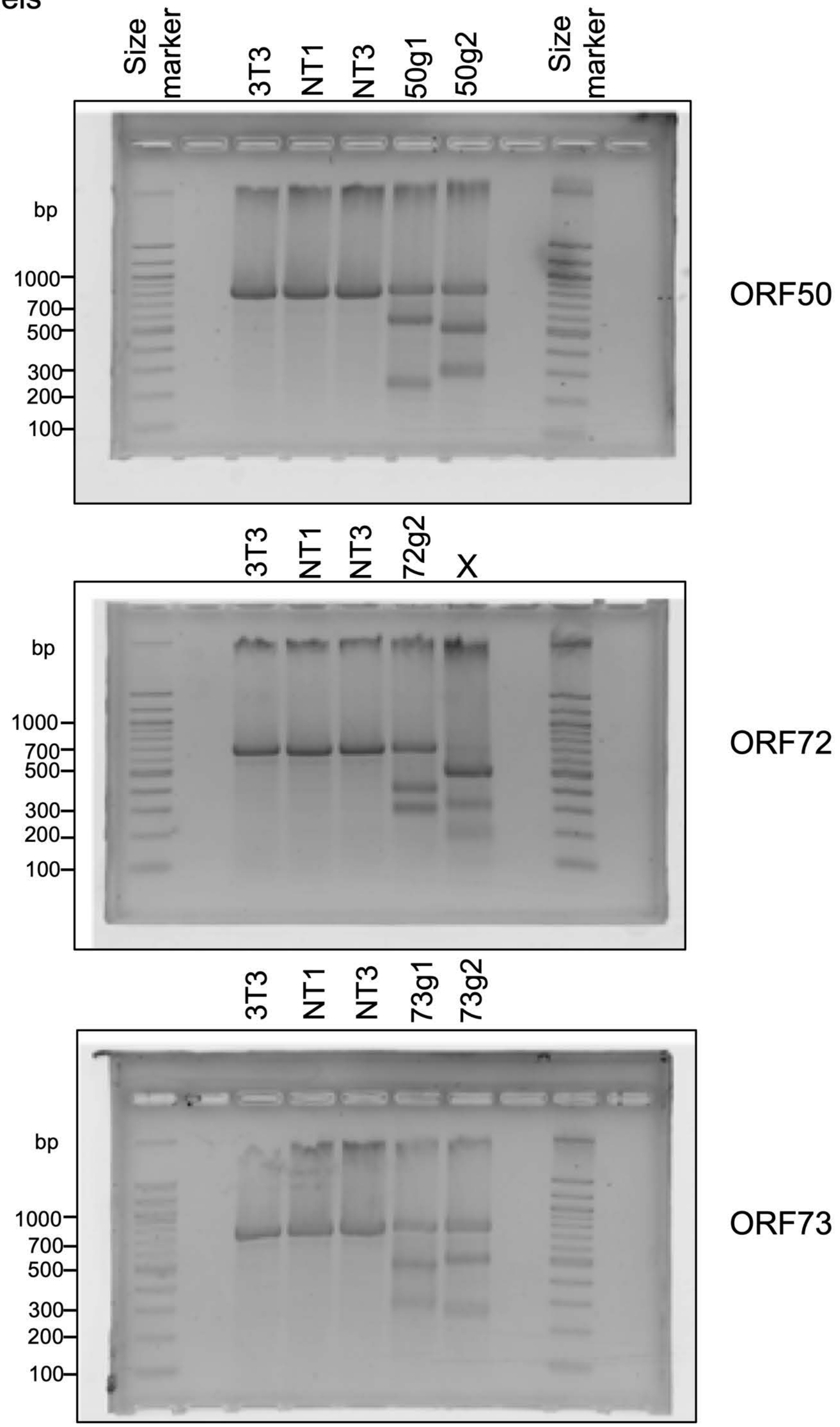

Final Figure 4B

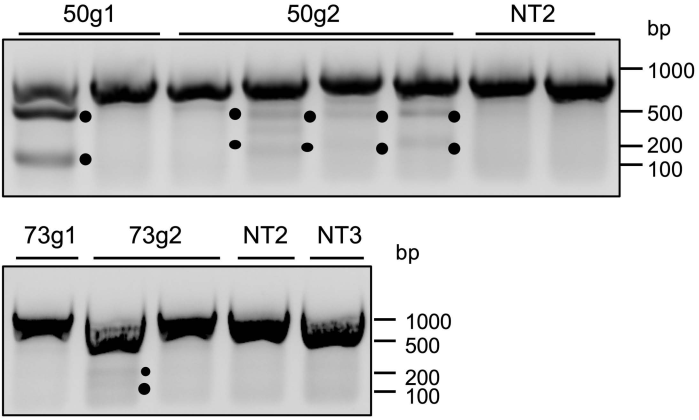

Original gel

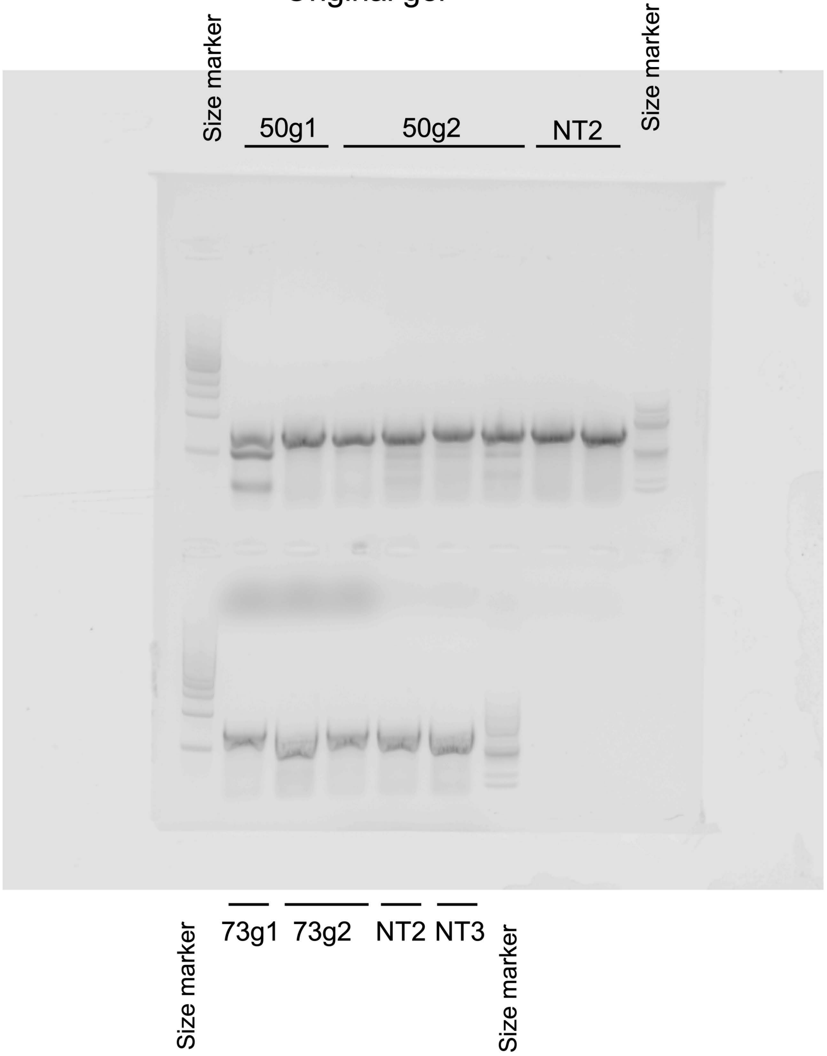

Supplement: S1 Raw images — Samples and size markers of the original blots or gels were labeled according to the descriptions of the final figures shown in the main text and unused lanes were indicated with “X”. (PDF) [file pone.0252313.s001.pdf]
